# Laser ablation electrospray ionization high‐resolution mass spectrometry for regulatory screening of domoic acid in shellfish

**DOI:** 10.1002/rcm.7725

**Published:** 2016-10-10

**Authors:** Daniel G. Beach, Callee M. Walsh, Pamela Cantrell, Wade Rourke, Sinead O'Brien, Kelley Reeves, Pearse McCarron

**Affiliations:** ^1^ Measurement Science and StandardsNational Research Council Canada1411 Oxford StreetHalifaxNSB3H 3Z1Canada; ^2^ Protea Biosciences Inc.1311 Pineview Dr.MorgantownWV26505USA; ^3^ Canadian Food Inspection Agency1992 Agency Drive, DartmouthNSB3B 1Y9Canada; ^4^ Marine InstituteRinvilleOranmoreCo. GalwayH91 R673Ireland

## Abstract

**Rationale:**

Domoic acid (DA) is a potent neurotoxin that accumulates in shellfish. Routine testing involves homogenization, extraction and chromatographic analysis, with a run time of up to 30 min. Improving throughput using ambient ionization for direct analysis of DA in tissue would result in significant time savings for regulatory testing labs.

**Methods:**

We assess the suitability of laser ablation electrospray ionization high‐resolution mass spectrometry (LAESI‐HRMS) for high‐throughput screening or quantitation of DA in a variety of shellfish matrices. The method was first optimized for use with HRMS detection. Challenges such as tissue sub‐sampling, isobaric interferences and method calibration were considered and practical solutions developed. Samples included 189 real shellfish samples previously analyzed by regulatory labs as well as mussel matrix certified reference materials.

**Results:**

Domoic acid was selectively analyzed directly from shellfish tissue homogenates with a run time of 12 s. The limits of detection were between 0.24 and 1.6 mg DA kg^−1^ tissue, similar to those of LC/UV methods. The precision was between 27 and 44% relative standard deviation (RSD), making the technique more suited to screening than direct quantitation. LAESI‐MS showed good agreement with LC/UV and LC/MS and was capable of identifying samples above and below 5 mg DA kg^−1^ wet shellfish tissue, one quarter of the regulatory limit.

**Conclusions:**

These findings demonstrate the suitability of LAESI‐MS for routine, high‐throughput screening of DA. This approach could result in significant time savings for regulatory labs carrying out shellfish safety testing on thousands of samples annually. © 2016 The Authors. Rapid Communications in Mass Spectrometry Published by John Wiley & Sons Ltd.

Domoic acid (DA; Fig. 1(A)) is a potent neurotoxin that is produced by marine diatoms and accumulates in shellfish. DA was first identified as the causative agent of amnesic shellfish poisoning (ASP) after a serious outbreak in 1987 that left 3 people dead after consuming contaminated blue mussels from Prince Edward Island, Canada.^1^ Since then, regulatory analysis of DA in shellfish has been critical in safeguarding public health and multi‐billion dollar shellfish industries worldwide, with the regulatory limit in Europe and North America set at 20 mg DA kg^−1^ edible tissue.^2^, ^3^

**Figure 1:**
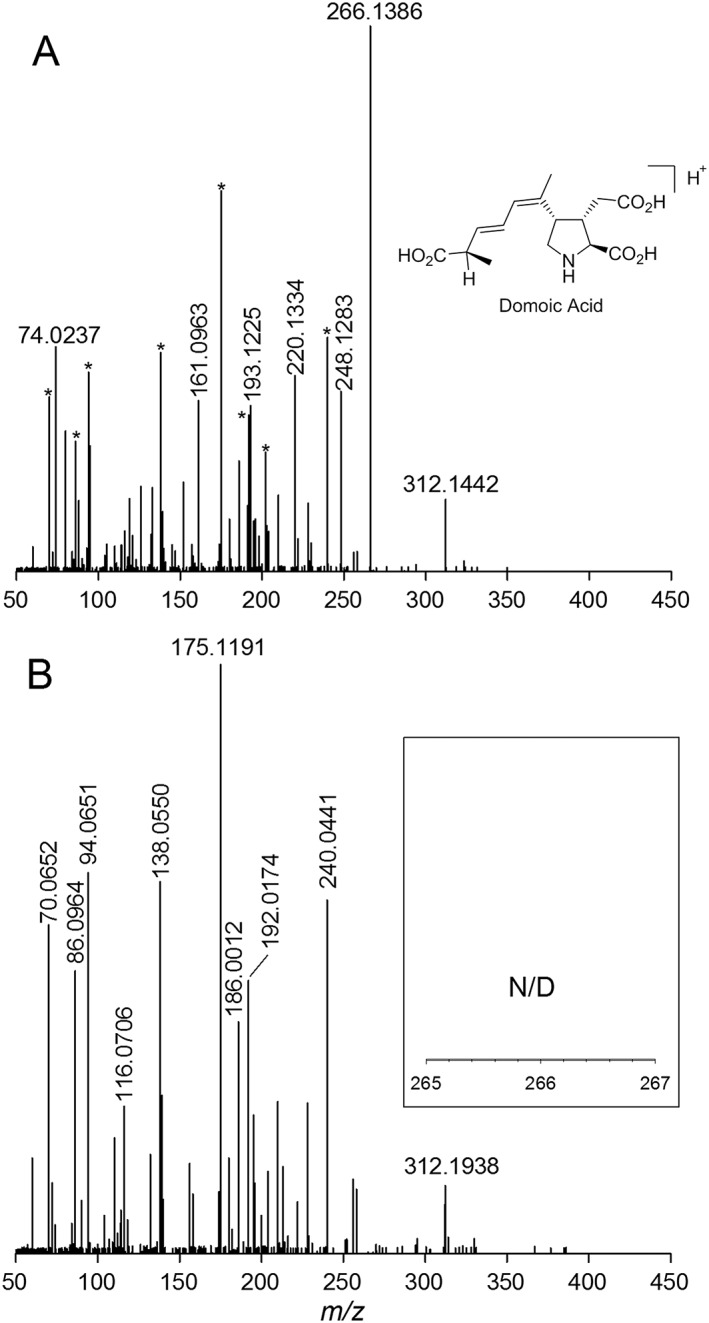
Selective detection of 20 mg kg^−1^ DA spiked into control mussel tissue homogenate using LAESI‐MS/MS of the precursor ion *m/z* 312 (A) and absence of significant matrix interference in analysis of un‐spiked control homogenate (B). Asterisks in (A) denote matrix background peaks present in control spectrum (B). Insets show an enlargement of the region around the *m/z* 266 product ion, which showed no detectable signal in the control.

Testing has typically been carried out using 10 to 30 min liquid chromatography/ultraviolet (LC/UV) methods after extraction with aqueous methanol, which are robust and fit for purpose.^4^, ^5^ Routine LC/mass spectrometry (MS) methods are also used for DA analysis, either as a single analyte or as part of a multi‐toxin analysis also capable of quantitating several classes of lipophilic toxins.^6^, ^7^ Routine LC/UV and dedicated LC/MS methods use external calibration with standards in neat solvent and are not significantly affected by matrix effects in electrospray ionization (ESI). More advanced LC/MS/MS methods are also available for DA, including sample preparation using strong anion‐exchange solid‐phase extraction (SPE)^5^ and/or derivatization chemistry,^8^, ^9^ that combined have yielded limits of detection as low as 1 μg kg^−1^.^10^ The scope of DA analysis worldwide is large enough that increases in sample throughput would lead to significant cost and time savings, particularly for regulatory labs. For example, the Canadian Food Inspection Agency (CFIA) and the Marine Institute (MI) in Ireland currently run about 10,000 and 3000 shellfish samples for DA testing annually, respectively, the majority of which are negative.

Efforts have already been made by regulatory labs to increase the throughput of DA analysis. The MI screens shellfish varieties other than scallops using a multi‐toxin LC/MS method with a limit of detection of 0.5 mg kg^−1^. Positive samples are then re‐analyzed using the regulatory LC/UV method,^11^ which is also used to analyze all scallop samples. The CFIA has developed rapid ultra‐high performance LC/UV and LC/MS methods, similar to that reported previously.^12^ These methods use a 2.5 min long chromatographic run and have been validated as fully quantitative with limits of detection (LODs) and limits of quantitation (LOQs) of 0.06 and 0.2 mg kg^−1^, respectively, for LC/MS and 0.7 and 2 mg kg^−1^, respectively, for LC/UV. Even compared with these more rapid methods, a screening method that does not require sample extraction or chromatographic separation would lead to a significant increase in sample throughput.

Screening samples for the presence or absence of a regulated substance using a high‐throughput technique followed by confirmatory analysis using a reference method has been recognized as an effective approach in many areas of food safety testing. The United States Department of Agriculture is currently moving towards LC/MS and gas chromatography (GC)/MS methods of high‐throughput screening for its analysis of chemical residues in meat.^13^ For algal toxins in shellfish, antibody‐based test kits have been considered for screening of paralytic poisoning toxins in shellfish by several regulatory agencies.^14^, ^15^ This assay has been reported to give a false positive rate of up to 30%, but still represented a significant benefit to the testing lab when used alongside the mouse bioassay as a confirmatory method.^14^ Recently, a comprehensive report compared the performance of five rapid test kits for qualitative and quantitative screening of DA in shellfish.^16^ Overall good performance was observed, with lateral flow immunoassays giving the best results. However, all these approaches still require sample extraction prior to analysis, which represents the practical limit to their throughput.

Laser ablation electrospray ionization (LAESI) is an ambient ionization technique for MS that uses a laser to produce a fine mist of neutral droplets of sample liquid followed by ionization of analytes by charge transfer from the plume of an electrosprayed buffer.^17^ During application of the laser pulse, water functions as a matrix, absorbing the mid‐infrared (λ = 2940 nm) laser energy by its O‐H stretch mode.^18^ The ionization specificity of LAESI is comparable with that of ESI rather than laser ablation techniques, making it more suitable for the analysis of polar, labile molecules. The majority of applications of LAESI have focused on mass spectrometry imaging of plant and animal tissues,^19^ but it has also been used for direct targeted and non‐targeted analysis in liquid samples and biological specimens.^20^, ^21^ To date, the capability of LAESI‐MS for targeted quantitative analysis directly from complex samples has remained largely unexplored.^20^, ^22^ Recently, we proposed that LAESI‐MS/MS could be used as a high‐throughput method for quantitative DA analysis and showed detection of DA directly from shellfish tissue homogenates.^22^ This preliminary proof of concept work considered only the blue mussel (*Mytilus edulus*) and only analyzed DA standards, spiked tissue and mussel tissue matrix reference materials. The LAESI‐MS/MS conditions were relatively generic and it was proposed that further refinement could lead to improvements in analytical variability.

Here, as a follow‐up to our original communication, we investigate the suitability of LAESI‐MS for analysis of DA in a wide range of real shellfish samples previously analyzed for DA in a regulatory setting. The first goal of this project was to refine the LAESI‐MS method for DA in shellfish tissue homogenates compared with the previous work,^22^ including transfer of the original method to a high‐resolution mass spectrometer for improved selectivity and sensitivity. Various sample preparation approaches and LAESI‐MS parameters were also optimized, with respect to the sensitivity of analysis, reproducibility and matrix effects. The second goal of this project was to evaluate the quantitative capabilities of the developed method for the analysis of real shellfish samples and to address the challenge of calibration using tissue homogenate standards. Finally, the quantitative results obtained by LAESI‐MS were compared with those obtained by standard LC/UV and LC/MS methods for a large set of shellfish samples.

## Experimental

### Samples and standards

A total of 189 shellfish samples analyzed previously as part of routine monitoring programs were acquired from the CFIA (Canada) and the MI (Ireland). The CFIA samples included 29 soft shell clam (Mya arenaria) samples ranging from 0.7 to 10 mg DA kg^−1^ wet tissue, five control (DA < LOD) clam samples and one blue mussel (Mytilus edulis) sample containing 14 mg DA kg^−1^ wet tissue. The MI samples included 13 king scallop (Pecten maximus) adductor muscle samples ranging from 0.8 to 16 mg DA kg^−1^ wet tissue and 13 control samples, 49 king scallop gonad samples ranging from 1.4 to 55 mg DA kg^−1^ wet tissue and five control samples, 23 king scallop remainder (viscera) samples with 0.6 to 222 mg DA kg^−1^ wet tissue and 38 blue mussels ranging from 2.8 to 21 mg DA kg^−1^ wet tissue, and 14 control samples. All samples were received as the homogenized tissue of several animals (≥100 g) from which sub‐samples for regulatory analysis had previously been taken.

NRC Certified Reference Materials (CRMs) for DA included a calibration solution (CRM‐DA‐f) and wet mussel matrix CRMs (CRM‐ASP‐Mus, CRM‐PSP‐Mus, CRM‐FDMT, CRM‐DSP‐Mus, NRC‐Zero‐Mus). The optimization of MS and LAESI parameters was carried out using a standard curve and mussel tissue homogenate matrix‐matched calibration standards prepared by spiking NRC CRM‐Zero‐Mus with a DA standard. The investigation of sample preparation approaches was then carried out by analyzing a sub‐set of real samples and in‐house reference standards prepared for this study. Matrix‐matched standards were prepared by blending naturally contaminated tissue with control tissue of each matrix. First, the control tissue was blended 1:1 with water and analyzed by LC/UV before DA was added. High‐level matrix‐matched standards were then prepared for each matrix by blending control tissue with ≤5% by mass of a high‐level, naturally contaminated, mussel tissue (>600 mg DA kg^−1^ tissue). A range of concentrations of matrix‐matched standards was then prepared by blending these high‐level samples with six different amounts of control tissue. Concentrations of DA were assigned to all these matrix reference materials using a validated LC/UV method.

### Optimized LAESI‐MS screening method

The test samples were diluted 1:1 with deionized water and further homogenized using an Omni TH handheld tissue homogenizer (Discovery Scientific, Vancouver, bc, Canada) to facilitate reproducible transfer of 20‐μL aliquots to low‐volume 96‐well plates using an automatic volumetric pipette with wide‐orifice 100‐μL tips. A LAESI DP‐1000 direct ionization system (Protea Biosciences Inc., Morgantown, WV, USA) was used to ablate samples with 50 pulses of a mid‐IR (λ = 2940 nm) laser at 10 Hz with 700 μJ of energy and a dwell time of 2 s. A Q Exactive hybrid Quadrupole‐Orbitrap mass spectrometer (Thermo Scientific, Waltham, MA, USA) was operated in in targeted positive ionization single ion monitoring (tSIM) mode with a 1 *m/z* unit window centred around *m/z* 312 (corresponding to the [M + H]^+^ ion of DA), at a mass resolution setting of *R* = 140 000 and a C‐trap fill time of 500 ms. The average MS peak height at *m/z* 312.144 across the LAESI peak was used to quantitate DA.

### Confirmatory LC/UV analysis

A validated LC/UV method was used to quantitate DA in the matrix standard reference materials prepared for this study and to re‐analyze some test samples after LAESI analysis. Sub‐samples (4 g) of homogenized mussel tissue were accurately weighed into a centrifuge tube with 16 mL of 1:1 methanol/water. These were vortex mixed for 3 min, centrifuged at 2600 *g* for 10 min and the supernatant was filtered to 0.45 μm prior to analysis. Determination of DA was carried out on an 1100 series LC system equipped with a diode array detector (Agilent, Palo Alto, CA, USA). Separation was carried out on a 3 μm Luna C18 stationary phase (150 x 4.6 mm column; Phenomenex, Torrance, CA, USA) with isocratic elution using 1:9 acetonitrile/water with 0.1% trifluoroacetic acid. The mobile phase flow rate was 0.9 mL min^−1^, the injection volume was 10 μL, the autosampler temperature was 6°C and detection was carried out at 242 nm.

## Results and Discussion

### LAESI‐MS method optimization

In our original communication, we used a linear ion trap (LIT) mass spectrometer to quantitate DA in MS/MS mode using the *m/z* 266 product ion obtained from the [M + H]^+^ ion at *m/z* 312.^22^ This method showed good selectivity and an LOD of 1 mg DA kg^−1^ in spiked wet mussel tissue homogenate. As reported previously, analysis of DA standards by LAESI‐MS and LAESI‐MS/MS gives spectra comparable with those generated by ESI.^22^ During method transfer to the Q Exactive (QE) instrument, we first compared sensitivity and selectivity between MS/MS and HRMS at various MS resolution settings using a control mussel tissue homogenate (CRM‐Zero‐Mus) spiked with DA at five levels between 1 and 60 mg kg^−1^. A product ion scan of the *m/z* 312 precursor showed excellent selectivity when mussel tissue homogenates containing DA were compared with the control homogenate (Fig. 1). Excellent mass accuracy of ±0.2 ppm for *m/z* 266 was also observed by MS/MS. However, only inconsistent detection of a few spectral counts was possible in the 1 mg kg^−1^ mussel matrix standard using MS/MS. The high selectivity of MS/MS resulted in no measurable background at the accurate mass of the *m/z* 266 product ion (Fig. 1(B)). This made it difficult to measure signal‐to‐noise ratios to determine an LOD, although this was estimated at about 2 mg kg^−1^. The drop in sensitivity in MS/MS mode observed between the LIT and the QE could be due to a combination of factors. The LAESI sampling capillary is longer on the QE than on the LIT by about 7.5 cm, which could lead to greater ion losses. In addition, the types of fragmentation techniques and ion detectors used on the two instruments are different, with the LIT using ion trap collision‐induced dissociation with an electron multiplier and the QE using higher energy collisional dissociation and Fourier transform detection. These methods have previously been shown to generate different data, both qualitatively and in terms of ion abundance.^23^

**Figure 2:**
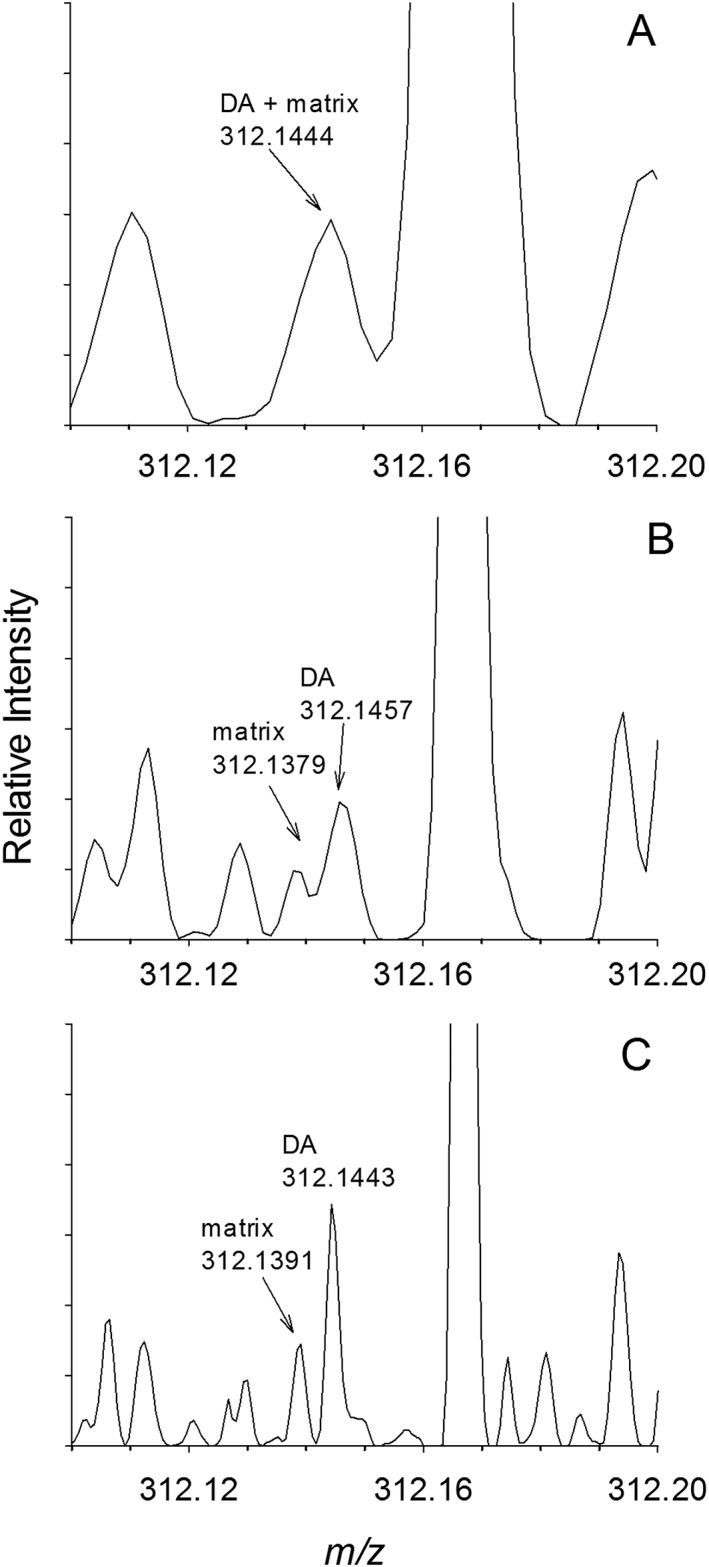
Isobaric matrix interference in LAESI‐MS analysis of DA spiked (1 mg kg^−1^) into control mussels at *R* = 35 000 (A) resolved by HRMS at *R* = 70 000 (B) and *R* = 140 000 (C).

Improved sensitivity and LODs were obtained by HRMS using the tSIM scan mode. This scan mode selects all ions of a specified nominal mass using the quadrupole for Orbitrap analysis as a way of minimizing space‐charge effects in the C‐trap and Orbitrap. The effect of resolution setting on selectivity and sensitivity in HRMS was also investigated using spiked mussel matrix‐matched calibration curves. The default setting of *R* = 35,000 gave a 10‐fold higher sensitivity than observed in MS/MS mode (Supplementary Fig. S1, Supporting Information). Only a modest drop in sensitivity of about 20% was observed when increasing resolution to *R* = 70 000, and no further decrease was observed using the highest resolution setting of *R* = 140 000. Increasing resolution showed a significant improvement in selectivity, as shown in Fig. 2. A single peak observed at *m/z* 312.1444 using *R* = 35 000 is partially resolved at R = 70 000, and completely resolved at R = 140 000, into a peak for DA at *m/z* 312.1443 and another for an interfering matrix species at *m/z* 312.1391. Using the instrument resolution setting of *R* = 140 000, the measured resolution for DA (*m/z*/FWHM) was 127 000 ± 2000 (*N* = 9) and the mass errors were ±1.3 ± 0.3 ppm (*N* = 15) in matrix samples (uncertainties given as standard deviation). This, along with the detection of significant intensities of this matrix interference in real mussel samples (discussed below), means that either a resolution setting of R = 140 000, used for the remainder of the current work, or MS/MS detection, used previously,^22^ is required for the selective analysis of DA from mussel tissue using LAESI‐MS techniques. Investigation of the matrix interference at *m/*z 312.1391 by LC/HRMS suggested that it was the M + 1 isotope peak of a matrix compound detected at *m/*z 311.1350. The matrix interference was not retained in reversed‐phase chromatography on a C_18_ stationary phase, and therefore does not represent a potential source of matrix interference in routine LC methods.

The LAESI pulse and the MS scan rate were synchronized by increasing the C‐trap fill time to 500 ms, which allowed for a larger number of laser pulses to be averaged in each MS scan and improved LAESI peak shape (Supplementary Fig. S2, Supporting Information). In order to maintain the number of data points per well at above 10, the number of pulses per well was increased from 30 to 50, all at a frequency of 10 Hz. To partially compensate for this decrease in throughput, the dwell time between wells was reduced from 5 s to 2 s.

After the experiments for the current study were complete, a follow‐up experiment was carried out on a prototype LAESI system capable of operating at laser pulse frequencies up to 20 Hz. A comparison of linearity and variability for a mussel tissue calibration curve run in triplicate at 10 Hz and 20 Hz showed improved linearity and a decrease in average RSD of replicate wells to 25% at 20 Hz, compared with 37% at 10 Hz (Supplementary Fig. S3, Supporting Information).

Early in method development, a trend in variable response with well position on the 96‐well plate was detected that showed significantly suppressed response from columns 1 and 2 of the 96‐well plate compared with other columns. This can be attributed to stage effects in the LAESI source: the disruption of the ion sampling due to air currents created by the moving stage. A larger impact of air currents on plume dynamics could be expected during the long stage movement from the last well of one row to the first well of the next (e.g. A12 to B1) compared with the short movements between wells in the same row (e.g. A11 to A12). At the time that the current experiments were being carried out, the LAESI stage was only able to move through wells in the sequence from left to right and return from column 12 to column 1 before beginning a new row (e.g. A1 to A12, B1 to B12, C1, etc.). In order to quickly eliminate this stage effect, columns 1 and 2 of each plate were not loaded with samples for the remainder of this study. In the time since these experiments were carried out, additional flexibility in stage movements has been accommodated in updated control software so that long stage movements can be avoided. It is expected that this will both correct the observed stage effect and increase throughput by eliminating the time required to scan back to the first well of the next row.

### Sample preparation optimization

**Figure 3:**
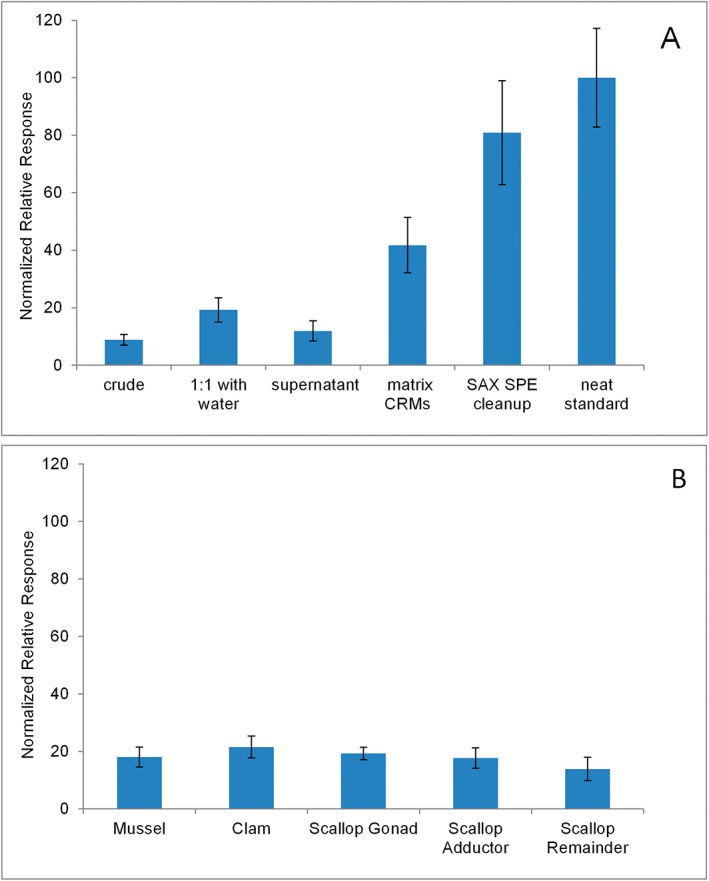
Sensitivity of analysis of DA in shellfish tissue homogenates using various sample preparation approaches (A) and in different tissue types (B). Data in (A) shows the average relative response of five real mussel samples or four reference materials normalized to the sensitivity of DA standards in solution. (B) shows the sensitivity of matrix standard curves, also normalized to the DA standard in (A). Error bars represent standard deviations of 3 to 5 samples.

Figure 3 shows a comparison of the sensitivity of analysis for DA in shellfish homogenates using different sample preparation approaches (Fig. 3(A)) and different tissue types (Fig. 3(B)). The normalized relative response plotted in Fig. 3 is defined as the MS spectral counts divided by the concentration of DA. This allowed the comparison of sensitivities between analyses of real samples in Fig. 3(A) and matrix standard curves in Fig. 3(B).

The low‐volume 96‐well plate format used for LAESI analysis requires reproducible dispensing of homogeneous 20‐μL aliquots of shellfish tissue homogenate. Additional sample homogenization was therefore required compared with what is typically required for LC analysis, where 2–5 g sub‐samples from larger batches of homogenized tissue are extracted with a 4:1 ratio of solvent/sample. To help with this additional homogenization and pipetting, the samples were diluted 1:1 with deionized water at this stage. For comparison, five samples from each tissue type were also analyzed without any further homogenization or dilution. Since it was not possible to pipette the crude tissue, small chunks were placed in each well using a spatula. Despite this practical limitation, DA was still successfully detected in positive samples, but with lower relative response than after dilution and further homogenization (Fig. 3(A)). It should be noted that the additional 1:1 dilution step required for LAESI‐MS analysis represents minimal additional work once incorporated into existing regulatory workflows, which already require some homogenization of several animals to account for biological variability.

Centrifugation of homogenates and analysis of crude supernatants by LAESI‐MS was also considered as a possible approach for sample preparation. In practice, this offered the ability to more reproducibly deliver 20‐μL aliquots into the low‐volume 96‐well plates. Analysis of supernatant by LAESI‐MS also represents a possible solution to the problem of storage and sub‐sampling of tissue homogenate standards needed to calibrate the LAESI‐MS method. It may not be practical to store a homogenate standard in the freezer, thawing it periodically to take a sub‐sample for LAESI calibration. By comparison, storage and sub‐sampling an aqueous supernatant is more feasible and similar matrix effects could be expected. Analysis of homogenate supernatants from the same sub‐set of samples as above showed a similar response and variability to the analysis of the diluted and blended samples (Fig. 3(A)). Since dilution and blending were found to be effective, centrifugation was not pursued further as an approach to sample preparation of the test samples, but it may still represent a reasonable solution to calibrating the method in the future.

Figure 3(A) also confirms the significant ionization suppression reported in the preliminary work, when the signal intensity from real samples was compared with that from neat solvent standards and samples fully extracted and cleaned‐up using strong anion‐exchange (SAX) solid‐phase extraction.^5^ While effective at improving sensitivity and minimizing matrix effects, the SAX cleanup is not compatible with a high‐throughput workflow and was only examined for comparison. Surprisingly, the response from NRC wet mussel tissue matrix CRMs was significantly higher than that of the raw shellfish samples, although the relative response between the CRMs was equivalent (Supplementary Fig. S4, Supporting Information). These materials have previously always been analyzed with methods requiring extraction and have excellent commutability with real samples with respect to matrix interference and ionization suppression when used in this way. Differences in processing between these CRMs and the real samples analyzed here include heat stabilization (cooking) and more rigorous homogenization, both of which could lead to differences in relative response in direct analysis by LAESI‐MS.

Differences in DA response and reproducibility, and matrix effects between the different shellfish tissue matrices were investigated and are shown in Fig. 3(B). The relative response between the different matrix calibration standards did not differ significantly, which is promising for use of a single matrix standard for calibration. In general, differences in matrix effects varied more between different sample preparation approaches than between the types of shellfish tissue being analyzed.

### Quantitative capabilities of the LAESI‐HRMS method

**Figure 4:**
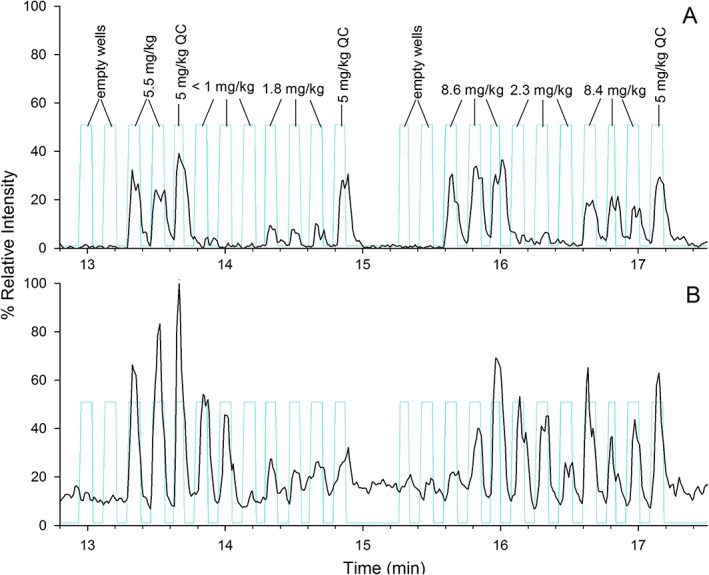
Selective detection of DA by LAESI‐HRMS at *m/z* 312.144 in mussel tissue homogenates (A) and resolution of an isobaric matrix peak at *m/z* 312.139 (B). Light blue traces indicate an analogue pulse used to approximate the time of each laser pulse and are annotated in (A) with DA concentrations of each sample determined from LC reference methods.

The quantitative capabilities of the developed LAESI‐HRMS method using the tSIM scan mode were assessed by investigating selectivity, linearity, LODs, precision and trueness. The reference materials included six‐point tissue homogenate matrix‐matched standards for each tissue type, with DA concentrations assigned by LC/UV. These were analyzed in at least triplicate, and the 5 mg kg^−1^ levels were analyzed at least 12 times throughout the run as quality control (QC) samples. The samples included 189 real shellfish samples previously analyzed by LC/UV or LC/MS as part of routine toxin monitoring programs. Samples and standards of each tissue type were analyzed together on 96‐well plates, with each sample spotted in triplicate. Figure 4(A) shows an example of the data generated from the analysis of a plate of mussel tissue homogenate samples.

The selectivity was assessed by confirming the absence of a signal at *m/z* 312.144 in 20 samples that tested negative for DA using LC reference methods. To investigate the relevance of the potentially interfering matrix species, discussed above and shown in Fig. 2, to the detection of DA in real samples, the accurate mass of the matrix interference at *m/z* 312.139 was determined from sequences of real shellfish samples. This matrix peak was absent in clam and scallop tissue samples, but was present at variable levels in mussels up to signal intensities equal to those of 10 mg kg^−1^ DA (Fig. 4(B)). Sample carryover was assessed by reviewing data from the analysis of real samples where low‐level samples followed high‐level samples. In one case (Supplementary Fig. S5(A), Supporting Information), significant sample carryover was observed after the analysis of a 0.5 mg kg^−1^ scallop adductor muscle sample immediately following a 40 mg kg^−1^ matrix standard. However, in nearly all other cases no carryover was observed. Supplementary Fig. S5(B) (Supporting Information) shows a near baseline signal for a 0.6 mg kg^−1^ scallop remainder sample when it was bracketed by the two highest level samples in the study: scallop remainder at 210 and 220 mg kg^−1^. The reason for the isolated carryover observed in Supplementary Fig. S5(A) (Supporting Information) is not clear, but care should be taken in manual review of data following high‐level detection.

The linearity was assessed from replicate analyses of six‐point matrix standards for each tissue type ranging from 1 to 40 mg kg^−1^. Considering the magnitude of matrix effects (Fig. 3), excellent linearity was observed for matrix‐matched calibration standards (Table 1), with R^2^ values ranging between 0.98 and 0.9992 (see also Supplementary Fig. S5, Supporting Information). The upper limit to the linear range was not investigated here but is not relevant to the use of LAESI‐MS in regulatory screening.

**Table 1:** Method performance results for DA analysis by LAESI‐HRMS in different shellfish tissue matrices

Tissue matrix	LOD^a^ (mg kg^−1^)	Precision of matrix standard %RSD	R^2^ of matrix‐matched calibration curve
Scallop adductor	0.50	27 (N = 13)	0.994
Scallop gonad	1.6	38 (*N* = 18)	0.98
Scallop remainder	0.62	38 (*N* = 12)	0.98
Clam	0.24	44 (*N* = 13)	0.9992
Mussel	1.1	36 (N = 22)	0.9991

a Limits of detection included the 1:1 dilution factor used in sample preparation.

Limits of detection of LAESI‐HRMS were determined through replicate analyses of low‐level (1–5 mg DA kg^−1^ wet tissue) matrix standards of each tissue type. This was done by calculating the signal‐to‐noise (S/N) as the ratio of the MS peak intensity at *m/z* 312.144 in low‐level matrix standards to that in control samples. These S/N values were then extrapolated to S/*N* = 3, doubled to account for the 1:1 dilution in sample preparation and used to determine LODs (Table 1). Similar values were obtained from the standard deviations of measurements on control samples, but an insufficient number of control samples for each tissue were available to allow rigorous calculation of LOD by this method. In all cases, samples close to the LOD as well as control samples were run to verify detection at these levels, which are similar to LODs of the LC/UV methods currently used for routine DA quantitation.^4^, ^5^, ^12^ Compared with other tissues, scallop gonad samples exhibited higher background and poorer LAESI peak shape, and these are reflected in a higher LOD.

The precision was assessed through replicate analysis of 5 mg kg^−1^ matrix reference standards of each tissue type run as QC samples throughout each sequence of real samples. These values (Table 1) are relatively high for quantitative analysis but generally acceptable for a screening method. They did, however, confirm that samples needed to be analyzed in at least triplicate to achieve good accuracy. Even with triplicate analyses, the run times were still about 30 s per sample.

**Figure 5:**
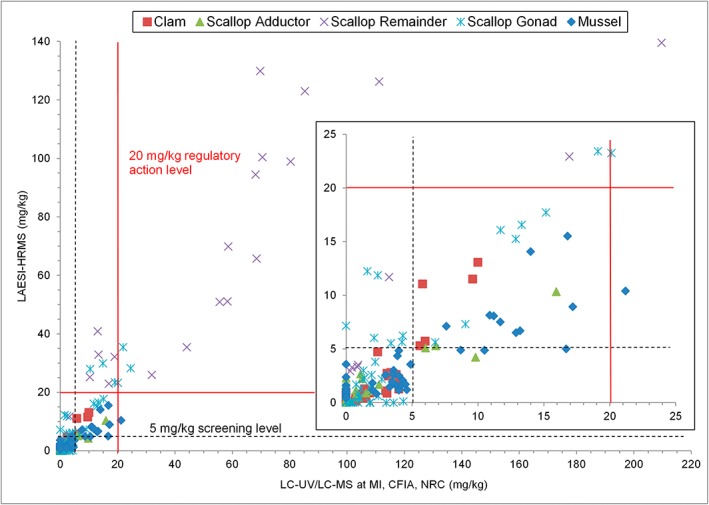
Comparison of LAESI‐HRMS screening using one‐point calibration with quantitation by validated routine LC/MS and/or LC/UV methods.

The accuracy of the method was evaluated by comparing results from LAESI‐HRMS with those from LC/UV and LC/MS reference methods. In addition, two different matrix‐matched calibration approaches were assessed using either single‐point calibration at 5 mg kg^−1^ or six‐point calibration from 1 to 40 mg kg^−1^. In all cases, the matrix‐matched standards consisted of shellfish tissue homogenates whose DA concentration was established using validated LC/UV methods. The results using the full calibration range were used to evaluate the performance of LAESI‐HRMS for primary quantitation. Single‐point calibration was used to evaluate its performance as a screening method. Two samples of scallop remainder with DA > 100 mg kg^−1^ showed saturation of detection and they were not considered when examining overall correlations. Both calibration approaches gave equal overall correlation coefficients (R^2^) of 0.89 for the linear regression of LAESI vs LC results. Six‐point calibration gave better overall agreement with a regression slope of 1.2 compared to 1.8 for single‐point calibration. This difference was primarily influenced by the highest level samples, which showed a significant positive bias using one‐point calibration. On the other hand, one‐point calibration gave better results for screening of samples as being either above or below 5 mg kg^−1^ (Fig. 5). Using this screening approach, LAESI‐HRMS was able to identify all 20 of the samples above the regulatory limit of 20 mg kg^−1^.^2^, ^3^ A false positive rate of 4% was observed, with eight samples being incorrectly identified as containing DA above 5 mg kg^−1^. Only one sample above 5 mg kg^−1^ was not identified by LAESI‐MS, but this sample was still found to contain less than half of the regulatory limit for DA, as determined by LC/UV.

The LAESI‐MS system was very robust and the experiments carried out for this study can be considered a significant stress test of the instrument. Approximately 2500 wells of shellfish tissue were analyzed over 2 days, which greatly exceeds sample volumes expected for routine use. It was found that the MS extension tube required cleaning after approximately 500 wells of tissue homogenate. This process involved sonicating the tube in a formic acid solution for 20 min and could be expected to have minimal impact on throughput as long as two tubes are available. The primary indication that extension tube cleaning was required was a drop in sensitivity, particularly for higher level samples, resulting in a loss of dynamic range of the method.

## Conclusions

LAESI‐HRMS performed well as a high‐throughput screening method for DA in a variety of shellfish matrices and was successful at identifying samples with [DA] >5 mg kg^−1^. This value corresponds to one‐quarter of the regulatory limit, and was chosen to allow for early detection of the beginning of an algal bloom event, to ensure no false negatives above 20 mg kg^−1^ and to minimize the number of false positives requiring confirmatory analysis by LC. No sample extraction or cleanup was required after the tissue had been homogenized. The analysis time, including replicates and standards required to obtain good trueness, was under 40 s/sample. Because of the high variability of the method, confirmatory testing of positive samples using an LC reference method is currently recommended. However, the availability of an isotopically labelled standard for DA in the future may allow LAESI‐HRMS to be used for direct quantitation without confirmatory testing.

Currently, the best approach for the calibration of LAESI‐MS is to prepare a secondary standard of homogenized, naturally contaminated, shellfish tissue, and to characterize it using a validated LC method. Remaining challenges include how to store and aliquot the shellfish homogenate standards required for LAESI‐MS calibration. Supernatants showed similar response to homogenates and could be useful as matrix‐matched standards in the future.

Use of this technique could result in significant cost and time savings for routine testing labs and expand their capacity during periods of unusually high sample volume, such as the *Pseudo‐nitzschia* bloom on the west coast of North America in 2015.^24^ The next stage of development should involve implementation of LAESI‐MS alongside current methodology in a regulatory testing lab where an ongoing comparison of the techniques could inform further method refinements and validation.

## Supporting information


**Fig. S1.** Comparison of sensitivity of different Orbitrap resolutions and scan modes for DA spiked control mussel tissue homogenates.
**Fig. S2.** Improved LAESI peak shape between 200 ms (A) and 500 ms (B) C‐trap fill times for DA‐spiked mussel tissue homogenates.
**Fig. S3.** Mussel tissue homogenate calibration standards analyzed by LAESI‐HRMS for 50 laser pulses at 10 Hz (A) and 100 laser pulses at 20 Hz (B). Error bars represent standard deviation of triplicate spotting at each level and numbers above each bar represent the percent standard deviation of the error bars.
**Fig. S4.** DA response in shellfish matrix. Error bars show standard deviation of *N* ≥ 3.
**Fig. S5.** Sample carryover observed from a high level matrix‐matched standard to a low level test sample in the LAESI‐MS analysis of DA in scallop adductor muscle tissue homogenate (A). Absence of sample carryover from high level to low level scallop remainder samples (B).

Supporting info item
