# Supplementary material for: Laser ablation electrospray ionization high‐resolution mass spectrometry for regulatory screening of domoic acid in shellfish
Source: Rapid Commun Mass Spectrom. 2016 Oct 10;30(22):2379–87. doi: 10.1002/rcm.7725 (PMC5434922; doi:10.1002/rcm.7725)
Supplement: Supplementary file 1 — Supporting info item [file RCM-30-2379-s001.docx]

Supporting Information

**Laser Ablation Electrospray Ionization-High Resolution Mass Spectrometry for Regulatory Screening of Domoic Acid in Shellfish**

Daniel G. Beach^a*^, Callee M. Walsh^b^, Pamela Cantrell^b^, Wade Rourke^c^, Sinead O’Brien^d^, Kelley Reeves^a^, Pearse McCarron^a^

a. Measurement Science and Standards, National Research Council Canada, 1411 Oxford Street, Halifax, NS, B3H 3Z1, Canada

b. Protea Biosciences Inc., 1311 Pineview Dr., Morgantown, WV, 26505 USA

c. Canadian Food Inspection Agency, 1992 Agency Drive, Dartmouth, NS, B3B 1Y9, Canada

d. Marine Institute, Rinville, Oranmore, Co. Galway, H91 R673, Ireland

*Corresponding author:

Dr Daniel G. Beach

Tel.: +1 (902) 426-8274; Fax: +1 (902) 426-9413

E-mail address: [daniel.beach@nrc-cnrc.gc.ca](mailto:daniel.beach@nrc-cnrc.gc.ca)

National Research Council of Canada

Measurement Science and Standards

1411 Oxford Street

Halifax, NS, B3H 3Z1, Canada

**Figure SI-1:** Comparison of sensitivity of different Orbitrap resolutions and scan modes for DA spiked control mussel tissue homogenates.

**Figure SI-2**: Improved LAESI peak shape between 200 ms (A) and 500 ms (B) C-trap fill times for DA-spiked mussel tissue homogenates.

**Figure SI-3**: Mussel tissue homogenate calibration standards analyzed by LAESI-HRMS for 50 laser pulses at 10 Hz (A) and 100 laser pulses at 20 Hz (B). Error bars represent standard deviation of triplicate spotting at each level and numbers above each bar represent the percent standard deviation of the error bars.

**Figure SI-4**: DA response in shellfish matrix. Error bars show standard deviation of N ≥ 3.

**Figure SI-5:** Sample carryover observed from a high level matrix-matched standard to a low level test sample in the LAESI-MS analysis of DA in scallop adductor muscle tissue homogenate (A). Absence of sample carryover from high level to low level scallop remainder samples (B).
